# Modification Mechanism of Glass Fibers on Ordinary Portland Cement and Sulphoaluminate Cement Composites

**DOI:** 10.3390/ma18081785

**Published:** 2025-04-14

**Authors:** Jiaxin Li, Tingquan Shao, Haoyan Guo, Zhenjun Wang, Ting Zhang, Jianliang Zhai, Yu Lei

**Affiliations:** 1 School of Materials Science and Engineering, Chang’an University, Xi’an 710061, China; 2 Shaanxi Union Research Center of University and Enterprise for Advanced Transportation Infrastructure Materials, Xi’an 710061, China

**Keywords:** ordinary Portland cement and sulphoaluminate cement composites, glass fiber, mechanical properties, drying shrinkage properties, pore structure

## Abstract

The problems of easy cracking, high brittleness, and low bond strength of ordinary Portland cement and sulphoaluminate cement (OPC-SAC) composites limit their application as rapid repair materials. In this study, glass fibers (GFs) were added to OPC-SAC composites with the content of 0.0–1.5% to improve their properties. Fluidity, mechanical properties, bond properties, and drying shrinkage properties were researched, and their microstructure was characterized by SEM and ICT. Hydration products at different curing ages were studied by XRD and FTIR. The results showed that GFs improved the mechanical properties of OPC-SAC composites. The 28 d flexural strength, compressive strength, and bond strength of specimens with 0.5% GFs reached maximum values, increasing by 22.1%, 12.1%, and 82.9%, respectively, compared with the control group without GFs.. GFs significantly inhibited the drying shrinkage of composites, and the inhibitory effect was magnified with the content of GFs. Adding 0.5% of GFs could reduce the porosity of specimens, decrease the volume proportion of pores (>10 mm^3^), and refine the pore structure. In summary, 0.5% is recommended as the optimal content of GFs to be added into the OPC-SAC composites.

## 1. Introduction

Sulphoaluminate cement (SAC) has been widely used in concrete repair projects for municipal, marine, and airport projects owing to its rapid hardening, high early strength, excellent corrosion resistance, and low energy consumption [1,2,3]. The key properties of SAC are primarily attributed to the presence of calcium sulfoaluminate ($C_{4}A_{3}\bar{S}$), dicalcium silicate (C_2_S), and calcium sulfate oxides. $C_{4}A_{3}\bar{S}$ is crucial for the rapid formation of ettringite (AFt), thereby accelerating setting and enhancing early strength development [4]. However, the short setting time and limited late-stage strength development, including issues such as reversed shrinkage, restrict its practical engineering applications. To address these limitations, researchers often incorporate ordinary Portland cement (OPC) into SAC to regulate the setting time and enhance mechanical properties [5]. Studies have shown that incorporating 10–20 wt.% OPC into SAC demonstrably enhances mechanical performance, resulting in more than a two-fold increase in compressive strength [6,7]. The enhanced crystallization stress of OPC-SAC composites relative to OPC arises from the higher supersaturation levels of AFt, which promotes expansive crystalline growth during hydration [8]. However, AFt is not sufficient to alleviate the shrinkage formed by cement at low SAC content [9]. OPC-SAC composites show a better corrosion resistance ability compared to OPC. OPC-SAC composites exhibit an optimal combination ability for Cl^−^, at an SAC content of 30% [10]. However, OPC-SAC composites still suffer from drawbacks such as cracking, high brittleness, and low bond strength, which limit their engineering applications.

The incorporation of steel fibers, basalt fibers, and lignin fibers in cement-based materials effectively addresses issues such as poor toughness, cracking, and low bond strength, while also improving long-term mechanical properties [11,12,13]. However, the limited corrosion resistance of steel fibers constrains their potential for enhancing the long-term crack resistance of cement-based materials [14]. Additionally, basalt and lignin fibers are prone to surface erosion and degradation in alkaline environments over extended periods, diminishing their toughening and reinforcing effects [15]. In contrast, glass fibers (GFs), as inorganic non-metallic materials with excellent chemical stability, environmental friendliness, and cost-effectiveness, are widely used to improve the deformation resistance and fracture toughness of cement-based materials [16,17]. Adding GFs to cement-based materials composed of OPC, SAC, and, coal gangue can significantly enhance the impermeability performance of specimens [7]. GFs can also reinforce the mechanical performance of cement, elevating tensile strength and toughness. Nevertheless, excessive amounts of GFs deteriorate the properties of the composites, which is mainly due to the disruption of the homogeneity of the matrix by the inhomogeneous distribution of fibers [18,19]. GFs act as microcrack bridges that can effectively inhibit stress propagation from crack tips, and thereby enhance durability [20]. However, research on the influence of GFs in OPC-SAC composites remains limited, particularly regarding their impact on the overall property of these composites. Therefore, it is necessary to evaluate the effect of GFs on the comprehensive property of OPC-SAC composites, determine the optimal content of GFs, and reveal the enhancement mechanism of GFs.

In this study, OPC-SAC composites with different GF contents were prepared. The mechanical properties, shrinkage properties, and workability of the composites were examined at different curing ages and GF doping levels. The mechanism of GFs in OPC-SAC composites was investigated using a combination of microscopic characterization techniques, including scanning electron microscopy (SEM), industrial computed tomography (ICT), X-ray diffraction (XRD), and Fourier transform infrared spectroscopy (FTIR). The results offer theoretical guidance for the application of GFs in OPC-SAC composite systems.

## 2. Experiments

### 2.1. Raw Materials

**Figure 1:**
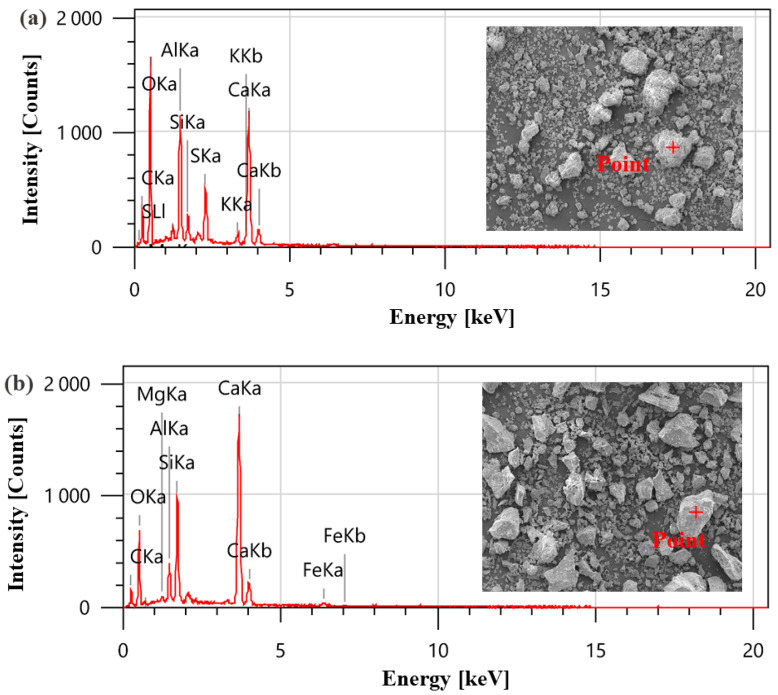
Microstructure and chemical composition of SAC and OPC: (**a**) SAC; (**b**) OPC.

**Figure 2:**
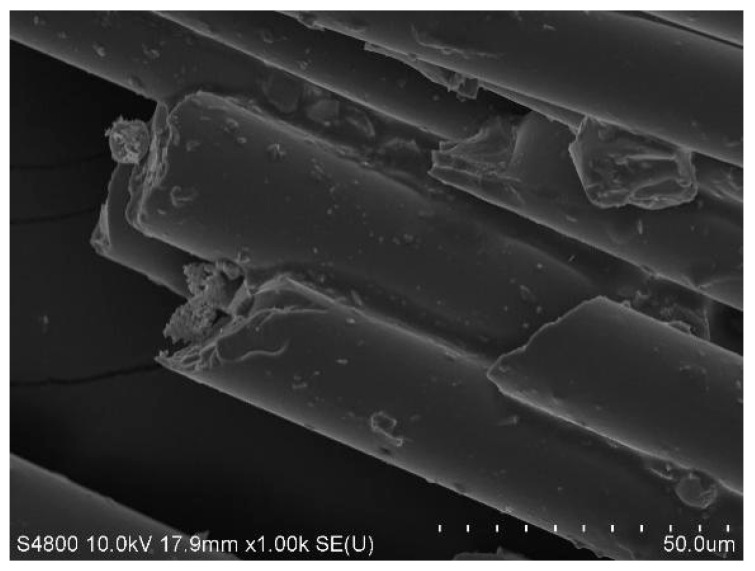
Microstructure of GFs.

In this study, OPC (P·O 42.5), SAC, ISO standard sand, and GFs were used. The chemical compositions of OPC and SAC were obtained by X-ray fluorescence (XRF-1800ASF(E), Shimadzu, Kyoto, Japan) and are presented in Table 1. Figure 1 characterizes the microstructural morphology and chemical composition of SAC and OPC. The physical properties and microstructure of GFs are detailed in Table 2 and Figure 2, respectively.

### 2.2. Specimen Preparation

**Figure 3:**
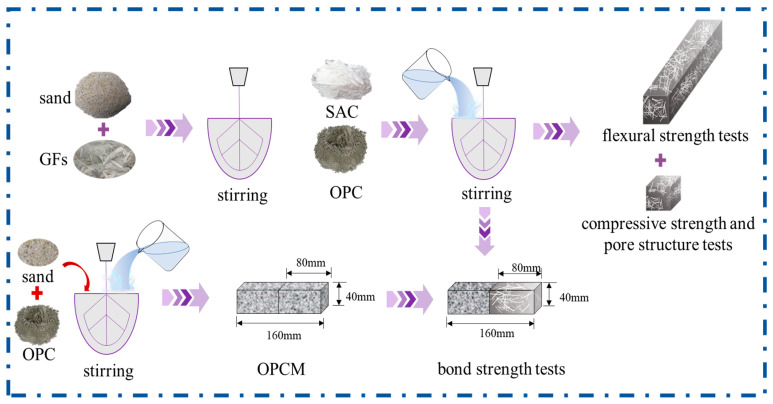
Sample preparation process diagram.

The specimen preparation process is illustrated in Figure 3. The mixture proportions are shown in Table 3. The cement mortar mixer (JJ-5, Jianyi Instruments & Machinery, Wuxi, China) was used. The GFs were mixed with sand and stirred, followed by the addition of OPC, SAC, and water, stirring for 2 min and then poured into molds, which were demolded after 5 h and then placed in a standard curing room. The fresh prepared mortars were transferred into molds for testing. The flexural strength of composite specimens cast in 40 mm × 40 mm × 160 mm molds was tested. The compressive strength and pore structure of composite specimens cast in 40 mm × 40 mm × 40 mm molds were tested. The dry shrinkage property of mortar specimens cast in 25 mm × 25 mm × 280 mm molds was tested.

The old Portland cement mortar matrix (OPCM) was formulated following the Chinese standard JTG 3420-2020 [21]. The OPCM after curing for 28 d was cut into 40 mm × 40 mm × 80 mm specimens, and the cutting surfaces were polished. The treated OPCM was placed on one side of the mold, and the fresh prepared mortar of OPC-SAC composites was poured into the other side to prepare the bond strength specimens. The specimens without sand were also prepared for SEM, XRD, and FTIR tests.

### 2.3. Test Methods

#### 2.3.1. Fluidity Test

The fluidity of the composites was evaluated based on the Chinese standard test method for the fluidity of cement mortar (GB/T 2419-2005) [22]. The mortar is loaded into the mold in two successive layers. The jumping table is immediately activated at a frequency of one cycle per second after the mold is vertically lifted, and is then deactivated after 25 cycles. The fluidity is defined as the average of the diameters measured along two mutually perpendicular directions on the bottom surface of the mortar.

#### 2.3.2. Mechanical Property Test

The flexural and compressive strengths of composites were tested according to the Chinese standard test method of cement mortar strength (GB/T 17671-2021) [23]. The tests were performed using a flexural and compressive testing machine (TYE-300E, Jianyi Instruments & Machinery, Wuxi, China). The flexural strength and compressive strength are determined by prismatic specimens (40 mm × 40 mm × 160 mm) and cubic specimens (40 mm × 40 mm × 40 mm), respectively.

#### 2.3.3. Bond Property Test

The bond property of the composites was evaluated in adherence to the Chinese standard repairing mortar (JC/T 2381-2016) [24]. The flexural strength of these bonded specimens was tested, and the interfacial bending and tensile strength results were expressed as the average flexural strength of six sets of specimens.

#### 2.3.4. Dry Shrinkage Property Test

The dry shrinkage property of composites (with dimensions of 25 mm × 25 mm × 280 mm) was assessed following the Chinese standard repairing mortar (JC/T 2381-2016) [24]. The initial shrinkage value was recorded after adding water for 3 h and demolding. Subsequently, the samples were placed in standard test conditions until specific ages (1 d, 3 d, 7 d, 14 d, and 28 d) were reached. The test result was determined as the average shrinkage value of the three groups of samples at specific ages.

#### 2.3.5. Microstructure Characterization

The microstructure of the composite paste was analyzed using SEM (S-4800, Hitachi, Tokyo, Japan). The internal pore structure of composites with different GF contents was examined using ICT (MultiscaleVoxel-2000, Sanying Precision Instruments, Tianjin, China). The analysis followed a three-step process: Firstly, ICT was employed to acquire a series of 2D images. Subsequently, the 3D reconstruction of the composites was performed using Voxel Studio Recon software (V2.2.3.6). Finally, the images were processed with non-local means filtering using Avizo software (2020.1), followed by threshold segmentation of the region of interest. The pores were analyzed to further obtain the internal pore structure and porosity of the sample. The hydration characteristics of the composite paste were investigated by XRD (D8 Advance, Bruker Corporation, Ettlingen, Germany). The instrument was equipped with a copper target and a ceramic X-ray tube, operating at 40 kV and 40 mA, with a 2θ scanning range of 15–80°. FTIR (BRUKER TENSOR II, Bruker Corporation, Ettlingen, Germany) was utilized to analyze the molecular structure and functional group composition of composite paste. The spectra were recorded in the 500–4000 cm^−1^ range.

## 3. Results and Discussion

### 3.1. Fluidity of Ordinary Portland Cement and Sulphoaluminate Cement (OPC-SAC) Composites Containing Glass Fibers (GFs)

**Figure 4:**
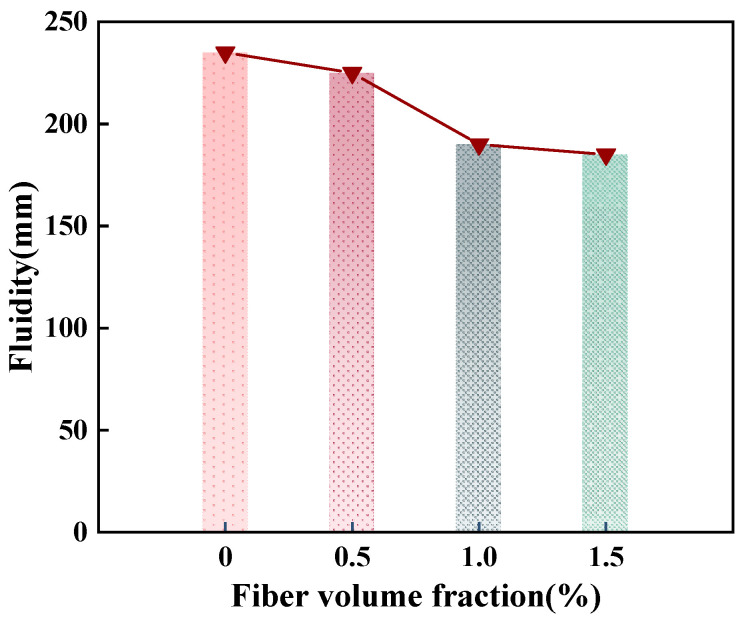
Fluidity of composites with different GF contents.

The fluidity of OPC-SAC composites with varying GF contents is illustrated in Figure 4. The mortar containing GFs has lower fluidity. The fluidity of GFs-0.5, GFs-1.0, and GFs-1.5 decreases by 4.3%, 19.2%, and 21.3%, respectively, compared to the Ref. specimen. The addition of GFs has a negative impact on the fluidity of the composites, owing to the increased friction between GFs and cement due to the random distribution of GFs.

### 3.2. The Mechanical Properties of Ordinary Portland Cement and Sulphoaluminate Cement (OPC-SAC) Composites Containing Glass Fibers (GFs)

#### 3.2.1. Flexural Strength

**Figure 5:**
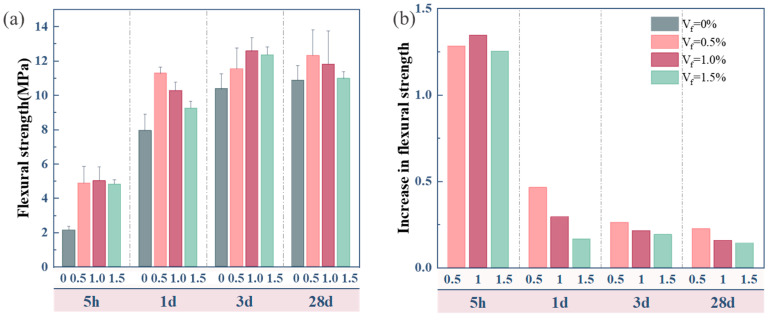
Flexural strength of composites: (**a**) flexural strength; (**b**) increase rate of flexural strength.

The flexural strength and variation trend of OPC-SAC composites with varying GF contents are illustrated in Figure 5. The composites containing GFs exhibit higher flexural strength compared to the Ref. specimen, as illustrated in Figure 5a. The flexural strength of composites shows a trend of first increasing and then decreasing with the increase in GFs content. GFs-0.5 reached 13.1 MPa and 13.3 MPa at the hydration curing age of 3 d and 28 d, respectively, which increased by 26.0% and 22.1% compared to composites without GFs. On the one hand, this is due to the fact that the appropriate volume of GFs is uniformly distributed in the composites and produces a toughening effect by forming a network structure. On the other hand, the anchoring effect between the GFs and matrix was enhanced due to the friction that prevents GFs from being peeled off from the cement matrix. However, the flexural strength of composites did not improve as the GFs content increased further. As the GFs content increases, the porosity of composites and the risk of bonding failure between the GFs and composites also increase. This is attributed to the phenomenon of agglomeration of GFs in composites caused by excessive fiber content [25]. The growth in the flexural strength of composites is shown in Figure 5b. The growth rate of flexural strength is larger at 1 d of hydration and relatively smaller after 3 d. This trend is related to the generation of AFt. The significant contribution of AFt to the development of early strength is due to its large generation in early stages of hydration.

**Figure 6:**
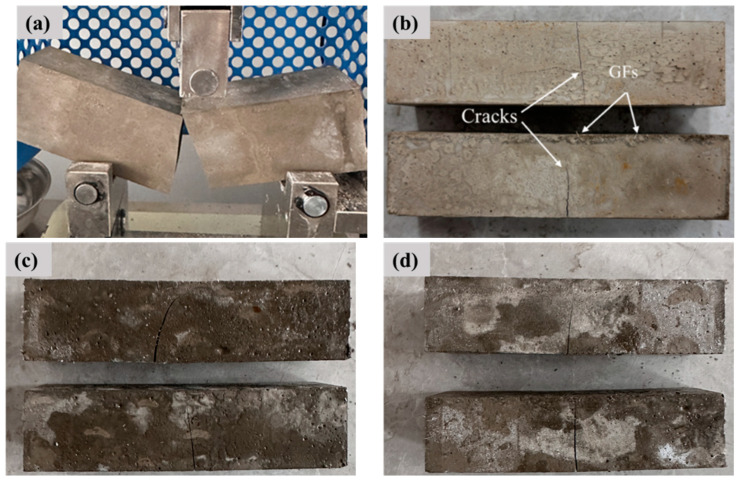
Morphological fracture of composites: (**a**) Ref.; (**b**) GFs-0.5; (**c**) GFs-1.0; (**d**) GFs-1.5.

The GFs content affects the fracture morphology of composites, as shown in Figure 6. The press stops immediately when the testing machine detects a sudden drop in load from the peak value. At this stage, typical brittle damage under bending loading is found in the Ref. specimen. The crack expanded rapidly and fractured into two parts after the Ref. specimen was subjected to loads. However, the composites containing GFs cracked and did not fracture completely after being loaded. This indicates that the specimen avoids direct fracture under loads due to the network toughness of GFs.

#### 3.2.2. Compressive Strength

**Figure 7:**
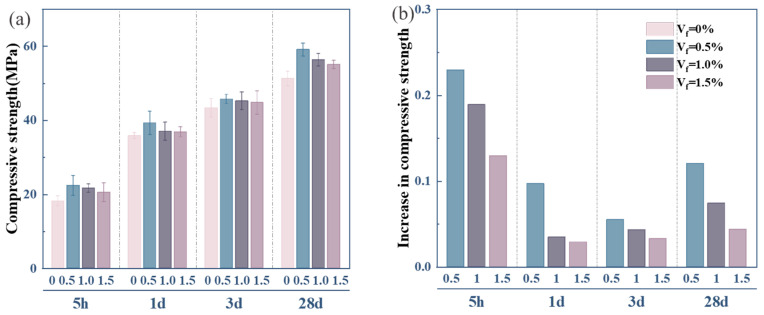
Compressive strength of composites: (**a**) compressive strength; (**b**) increase rate of compressive strength.

Figure 7 presents the compressive strength of OPC-SAC composites. As shown in Figure 7a, the composites doped with GFs have a higher strength compared to the Ref. specimen. The 3 d and 28 d compressive strengths of GFs-0.5 reach 45.7 and 59.1 MPa, respectively. The compressive strength can be enhanced by 0.5% GFs, owing to the random distribution of GFs within the composites. Additionally, the optimization of the pore structure can provide additional load-bearing capacity to the composites. Appropriate amounts of GFs are tightly embedded with hydration products during cement hydration to form a network structure. This network structure is capable of transferring and distributing the applied loads efficiently, further enhancing the compressive strength. The network structure of GFs restrains the lateral strain of composites. However, the excessive GFs addition will trigger a trend of decreasing the compressive strength of composites. The phenomenon of the rapid increase in early strength can be observed in Figure 7b. In addition, the growth rate of compressive strength at 28 d of hydration is increased compared to that at an early stage. The early-stage strength development of the composites is predominantly governed by the rapid hydration of SAC. This initial phase is characterized by the accelerated nucleation and growth of AFt, which impart critical mechanical strength through their needle-like morphology. The mineral C_3_S in OPC has not fully hydrated within 3 d. The secondary hydration reaction of OPC is significantly enhanced after 28 d. The density of the composites’ matrix increases due to the gradually generated C-S-H gel filling pores.

### 3.3. Effect of Glass Fiber (GF) Contents on Bond Property Between Composites and Old Portland Cement Mortar Matrix (OPCM)

**Figure 8:**
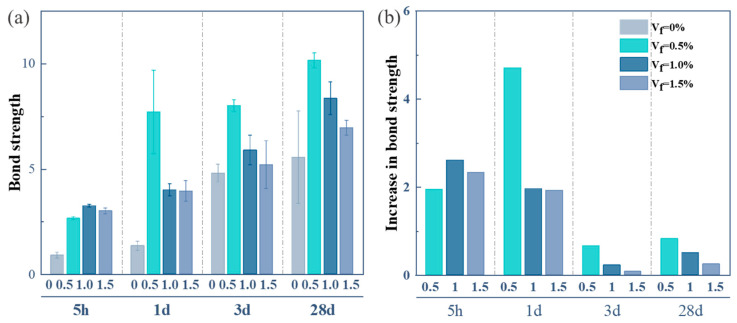
Bond strength between composites and OPCM: (**a**) bond strength; (**b**) increase rate of bond strength.

Figure 8 illustrates the bond strength between OPC-SAC composites and OPCM. As shown in Figure 8a, composites incorporating GFs demonstrate a higher bond strength than the Ref. Figure 8b further reveals that the bond strength of GFs-0.5, GFs-1.0, and GFs-1.5 specimens increases by 82.9%, 50.4%, and 25.2%, respectively, compared to the Ref. Among these, the GFs-0.5% specimen exhibits the highest bond strength. This enhancement can be attributed to the hydrophobicity of GFs. It is determined by the stress transmission of GFs and the crack suppression effect. In addition, the distribution of 0.5% GFs in the mortar is uniform, which can effectively transmit stress and suppress the generation and propagation of cracks [26].

### 3.4. Effect of Glass Fiber (GF) Contents on the Shrinkage Properties of Ordinary Portland Cement and Sulphoaluminate Cement (OPC-SAC) Composites

**Figure 9:**
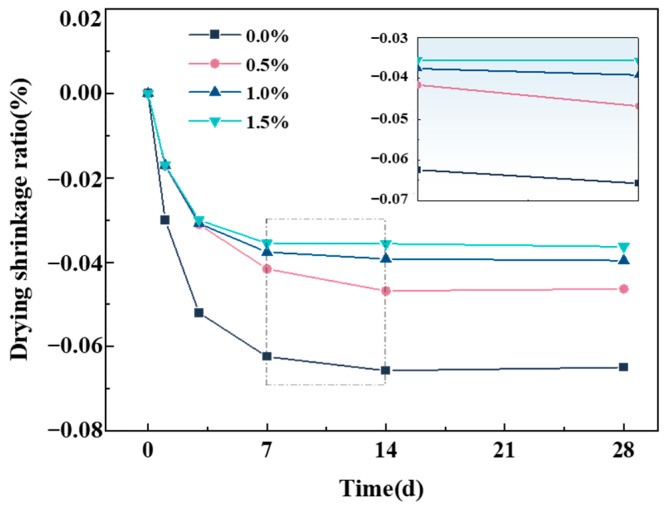
Drying shrinkage of OPC-SAC composites.

Figure 9 illustrates the shrinkage behavior of cement with varying GF contents. The shrinkage process of composites consists of two distinct stages. In the first stage, a significant shrinkage rate can be observed within the initial 7 d of hydration. In the second stage, the shrinkage rate gradually stabilizes after 7 d of hydration. The 7 d shrinkage rates of GFs-0.5, GFs-1.0, and GFs-1.5 specimens decrease by 33.3%, 39.7%, and 43.1%, respectively, compared to the Ref. specimen. As the GFs content increases, the shrinkage rate of composites progressively decreases, and the shrinkage inhibition effect becomes more pronounced. The shrinkage inhibition of cement by GFs is primarily attributed to two mechanisms: Firstly, a mechanical interlocking effect occurs between GFs and the cement matrix. Randomly distributed GFs form a skeletal structure within the cement, effectively restraining the development of internal tensile stress, and thus reducing shrinkage deformation [27]. Secondly, GFs dispersed within the matrix hinder moisture transport, slow the rate of water loss, and consequently suppress cement shrinkage [28]. Increasing the GFs content reduces fiber spacing, further constraining cement shrinkage deformation. Therefore, the higher GFs content leads to a more pronounced shrinkage inhibition effect within a certain range.

### 3.5. Microstructure of Ordinary Portland Cement and Sulphoaluminate Cement (OPC-SAC) Composites

#### 3.5.1. Glass Fibers (GFs)–Matrix Interface Microstructure

**Figure 10:**
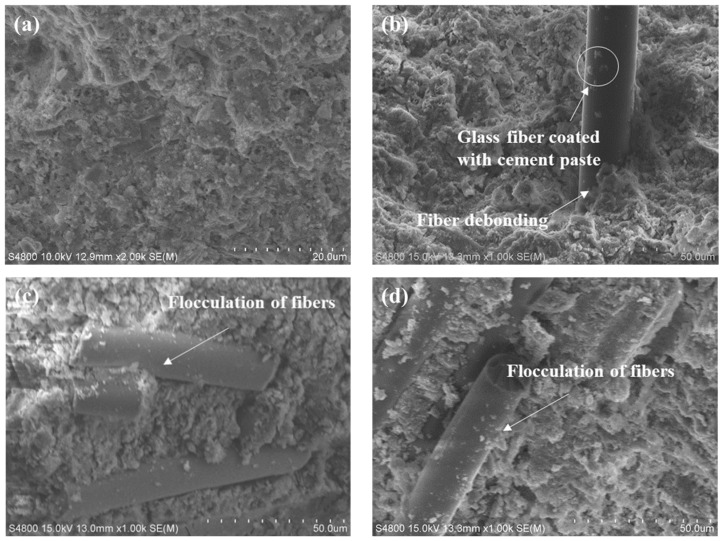
Microstructure of GFs–matrix interface at 3 d of curing ages: (**a**) Ref.; (**b**) GFs-0.5; (**c**) GFs-1.0; (**d**) GFs-1.5.

Figure 10 illustrates the microstructure of the GFs–matrix interface with varying GF contents. As observed in Figure 10a, the microstructure of composites without GFs appears denser. In Figure 10b–d, the microstructures of the interface between GFs and the cement matrix at 0.5%, 1.0%, and 1.5% GF contents exhibit strong interfacial adhesion, because the GFs are inorganic non-metallic mineral fibers with excellent hydrophilicity [29]. Moreover, the microstructure of the cement matrix becomes progressively more porous as the GFs content increases. In Figure 10b, the surface of the GFs is covered with hardened matrix hydration products, which can enhance the bond strength between the matrix and fibers. The random distribution of GFs within the composites’ matrix effectively prevents crack propagation, which explains the improvement in mechanical properties at low GFs content. However, when the GFs content exceeds 0.5%, the uneven distribution and agglomeration of GFs become the primary causes of the decline in both mechanical and bond properties of the composites. Figure 10b–d also show that the hydration products of composites adhere on the surface of the GFs, indicating that there is a compatibility between the matrix and GFs. Meanwhile, the fibers are not broken but pulled out when the cement matrix is damaged. Additionally, the randomly dispersed GFs interlock uniformly and effectively with the hydration products, which can disrupt the original stress distribution within the cement, thereby reducing shrinkage deformation.

#### 3.5.2. Pore Structure of Ordinary Portland Cement and Sulphoaluminate Cement (OPC-SAC) Composites

**Figure 11:**
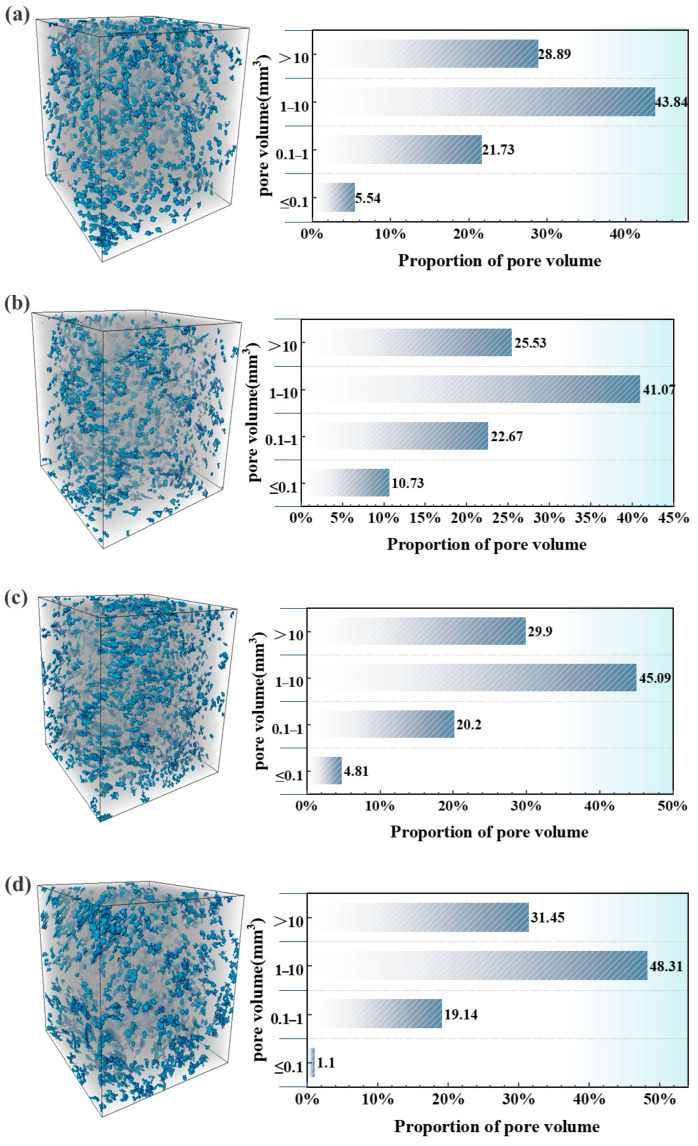
Proportion of pore volume of composites at 28 d: (**a**) Ref.; (**b**) GFs-0.5; (**c**) GFs-1.0; (**d**) GFs-1.5.

The microstructure of OPC-SAC composites with varying GF contents is observed using ICT, and then reconstructed by AVIZO to calculate the pore volume occupancy. The results are shown in Figure 11. Typically, the internal pores of the cement matrix can be categorized into four distinct size ranges [30]: >10 mm^3^, 1–10 mm^3^, 0.1–1 mm^3^, and ≤0.1 mm^3^. As illustrated in Figure 11, the pore distribution within the cement matrix is predominantly characterized by pores with a volume ranging from 1 mm^3^ to 10 mm^3^. The percentage of pores with a volume > 10 mm^3^ of GFs-0.5 was reduced by 11.6% compared to the Ref. specimen, indicating that a refinement of pore structure occurred due to the addition of GFs. Similarly, the 1–10 mm^3^ pore reduction in GFs-0.5 further corroborates the refining effect of GFs on the pore structure of the matrix, thereby enhancing the mechanical strength. As demonstrated in Figure 11c,d, the GFs-1.0 and GFs-1.5 samples exhibited 3.5% and 8.9% increases in pore volume (>10 mm^3^) relative to the Ref. specimen.

The effect of the GFs content on the porosity of the cement matrix is outlined in Table 4. There is a slight increase in the porosity of the composites as the content of GFs increased, and the ranking results are as follows: GFs-1.5 > GFs-1.0 > Ref. > GFs-0.5. It has also been shown that the incorporation of GFs impacts the porosity of the cement matrix and enhances its resistance to cracking [31,32]. This ability of GFs to resist the expansion of cracks within the composites is also responsible for the increase in the compressive strength of the composites. Furthermore, the variance is ranked as GFs-1.5 > GFs-1.0 > Ref. > GFs-0.5. The porosity variance indicates pore distribution within the matrix, with a smaller variance suggesting more uniform dispersion [33]. The moderate addition of GFs (GFs-0.5) can reduce cement porosity and promotes uniform pore distribution.

#### 3.5.3. Hydration Products of Ordinary Portland Cement and Sulphoaluminate Cement (OPC-SAC) Composites

**Figure 12:**
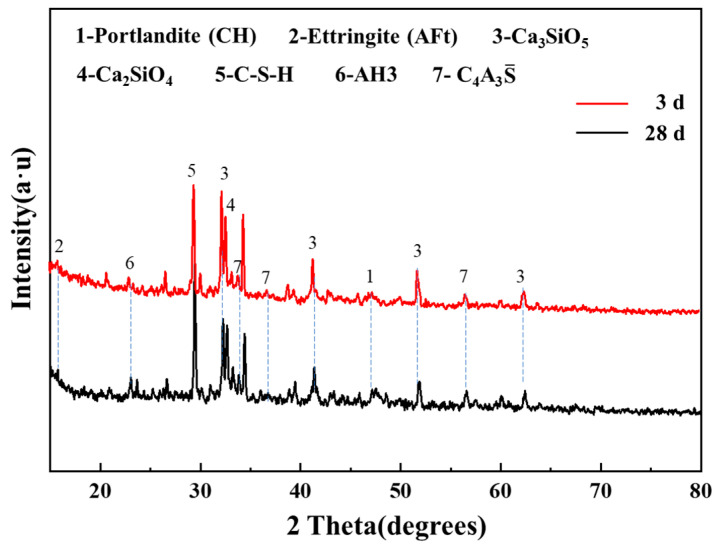
XRD patterns of OPC-SAC composites.

Figure 12 presents the XRD pattern of OPC-SAC cement paste, in which the diffraction peaks of CH, AFt, C_3_S, C_2_S, C-S-H, AH_3_, and C_4_A_3_$\bar{S}$ are clearly discernible. The XRD analysis revealed prominent peaks at 29.1°, 15.78°, 41.3°, 32.53°, 23.35°, and 56.5° in OPC-SAC composites, corresponding to characteristic crystalline phases of CH, AFt, C_3_S, C_2_S, AH_3_, and C_4_A_3_$\bar{S}$, respectively [34,35,36]. Significantly, AFt is recognized as the predominant hydration product at early stage of cement hydration. When SAC is mixed with water, $C_{4}A_{3}\bar{S}$ reacts rapidly to produce AH_3_ and AFt [37], which rapidly form and fill the pores, providing satisfactory early strength to the cement. In comparison to $C_{4}A_{3}\bar{S}$, C_2_S hydrates more gradually and hydrates to form C-S-H gels and CH, thereby leading to the denser cement structure [38]. As illustrated in Figure 12, the intensity of the diffraction peaks of AFt at 28 d is higher than 3 d. This phenomenon is attributed to the combination of CH generated by C_2_S hydration with C_4_A_3_$\bar{S}$ and gypsum to produce AFt [39]. The presence of OPC is vital for the hydration of SAC cement. The CH diffraction peak is not readily apparent at 3 d of cement hydration, while its visibility increases at 28 d, attributable to two factors. First, the delayed hydration of C_2_S and C_3_S results in a reduced yield of CH. Secondly, the CH hydration reaction that produces AFt is responsible for the continuous increase in cement strength. The XRD spectrum shows a broad featureless hump centered at 2θ = 29°. It indicates that amorphous C-S-H gels are observed. The diffraction peak of C-S-H shifted to a higher angle and the intensity increased at 28 d. This indicates that C-S-H partially hydrates within 3 d and continues to hydrate in the later stages. This is the reason why the compressive strength increased later.

**Figure 13:**
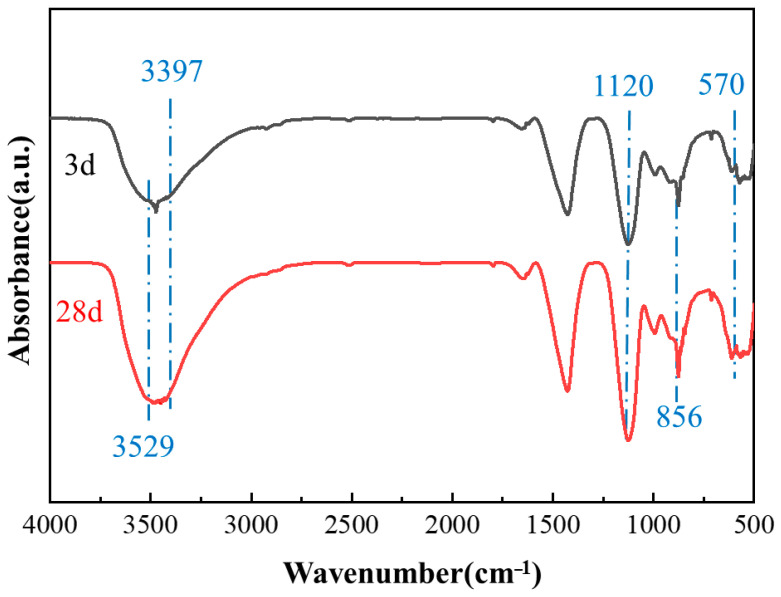
FTIR spectra of OPC-SAC composites.

The FTIR spectra of the composites for different curing times are shown in Figure 13. The absorption peak at 570 cm^−1^ corresponds to the [Al$O_{6}^{-}$] octahedral structural absorption peak of the AH_3_, while the absorption peak at 856 cm^−1^ exhibits an asymmetric telescoping vibration of AlO_4_^5−^ in the SAC. In addition, the absorption peak at 3400 cm^−1^ is attributed to the O-H bond stretching vibration of the C-S-H gel water of crystallization. In particular, the peak at 3400 cm^−1^ at 28 d was smaller than at 3 d, indicating the slower hydration of OPC cement than SAC. The FTIR spectra of cement hydration at 28 d show a stronger peak centered at 1120 cm^−1^, attributed to the high frequency of asymmetric telescopic vibrations of columellate. Additionally, the width of the gypsum band in the range of 3397–3529 cm^−1^ for 28 d of hydration was observed to be weaker in comparison to the 3 d hydration period, thereby suggesting that C_4_A_3_$\bar{S}$ hydrated with CH and gypsum.

## 4. Conclusions

This work investigated the effects of different contents of GFs on the fluidity, mechanical, and shrinkage properties of OPC-SAC composites. The main conclusions are as follows:

(1) The moderate content of GFs can improve the mechanical properties of cement. The flexural strength, compressive strength, and bond strength of OPC-SAC composites reach the maximum at 0.5% GFs content. This phenomenon is attributed to the ability of appropriate amounts of GFs to be embedded in the hydration products of the composites, forming a network structure.

(2) The incorporation of GFs effectively inhibits the composites’ shrinkage, and the inhibiting effect on shrinkage becomes more pronounced as the GF contents increase. This is attributed to the fact that the skeletal structure of GFs enhances the binding force of cement and effectively improves the shrinkage property of the composites.

(3) At 0.5%, GFs are uniformly distributed to form a network structure and improve the pore structure of the composites. The incorporation of 0.5% GFs reduces the percentage of pores with a volume > 10 mm^3^ and the porosity of the composites. GFs are unevenly distributed and agglomerated in the matrix when the its content exceeds 0.5%.

(4) The high early strength of the composites is attributed to the rapid reaction of calcium sulphoaluminate with calcium silicate to form AFt. The sustained increase in the composites’ strength at later stages is due to the reaction of calcium sulphoaluminate, calcium hydroxide, and gypsum.

## 5. Further Research

The distribution of internal pores is influenced by the distribution of fibers. Therefore, the distribution and orientation of fibers in the matrix should be further quantitatively calculated and visualized in the future. The quality of the adhesion between the fibers and matrix is influenced by various factors such as the distribution, length to diameter ratio, and thickness of the fibers. The influence of these parameters of fibers on the adhesion quality between the fibers and matrix should be further considered.

## Figures and Tables

**Table 1:** Chemical composition of raw materials (wt.%).

Constituents	SiO_2_	Al_2_O_3_	CaO	MgO	Fe_2_O_3_	SO_3_	Others
SAC	12.3	27.5	43.6	1.6	1.9	11.5	1.6
OPC	18.4	5.2	64.5	1.3	3.4	2.1	5.1

**Table 2:** The physical properties of GFs.

Properties	Diameter (μm)	Length (mm)	Tensile Strength (MPa)	Elastic Modulus (GPa)	Specific Gravity (g·cm^−3^)
GFs	12.00	12.00	1700.00	70.00	2.180

**Table 3:** Mixture proportions of OPCM and OPC-SAC composites.

Group	Mass Ratio (%)		Cement/Sand Ratio	W/C	Volume Fraction of GFs (%)
	OPC	SAC			
OPCM	1.0	0	1:3	0.5	0
Ref.	0.2	0.8	1:2.5	0.5	0
GFs-0.5	0.2	0.8	1:2.5	0.5	0.5
GFs-1.0	0.2	0.8	1:2.5	0.5	1.0
GFs-1.5	0.2	0.8	1:2.5	0.5	1.5

**Table 4:** Porosity and variance of cement composites with different GF contents.

Specimen	Ref.	GFs-0.5	GFs-1.0	GFs-1.5
Porosity (%)	8.75	8.17	9.05	9.34
Variance	0.71	0.63	0.78	0.87

## Data Availability

The original contributions presented in this study are included in the article. Further inquiries can be directed to the corresponding authors.
